# S-thanatin functionalized liposome potentially targeting on *Klebsiella pneumoniae* and its application in sepsis mouse model

**DOI:** 10.3389/fphar.2015.00249

**Published:** 2015-10-27

**Authors:** Xiaobo Fan, Juxiang Fan, Xiyong Wang, Pengpeng Wu, Guoqiu Wu

**Affiliations:** ^1^Center of Clinical Laboratory Medicine of Zhongda Hospital, Southeast UniversityNanjing, China; ^2^Medical School, Southeast UniversityNanjing, China

**Keywords:** targeting delivery, sepsis, antimicrobial peptide, liposome, multidrug resistance

## Abstract

S-thanatin (Ts) was a short antimicrobial peptide with selective antibacterial activity. In this study, we aimed to design a drug carrier with specific bacterial targeting potential. The positively charged Ts was modified onto the liposome surface by linking Ts to the constituent lipids via a PEG linker. The benefits of this design were evaluated by preparing a series of liposomes and comparing their biological effects *in vitro* and *in vivo*. The particle size and Zeta potential of the constructed liposomes were measured with a Zetasizer Nano ZS system and a confocal laser scanning microscope. The *in vitro* drug delivery potential was evaluated by measuring the cellular uptake of encapsulated levofloxacin using HPLC. Ts-linked liposome or its conjugates with quantum dots favored bacterial cells, and increased the bacterial uptake of levofloxacin. In antimicrobial assays, the Ts and levofloxacin combination showed a synergistic effect, and Ts-LPs-LEV exhibited excellent activity against the quality control stain *Klebsiella pneumoniae* ATCC 700603 and restored the susceptibility of multidrug-resistant *K. pneumoniae* clinical isolates to levofloxacin *in vitro*. Furthermore, Ts-LPs-LEV markedly reduced the lethality rate of the septic shock and resulted in rapid bacterial clearance in mouse models receiving clinical multidrug resistant (MDR) isolates. These results suggest that the Ts-functionalized liposome may be a promising antibiotic delivery system for clinical infectious disorders caused by MDR bacteria, in particular the sepsis related diseases.

## Introduction

Increasing data from clinics has revealed the wide spreading of multidrug resistant (MDR) *Klebsiella pneumoniae* resistant to almost all conventional antibiotics with different structures, such as cephalosporins, carbapenems, fluoroquinolones, lincosamides, and aminoglycosides (Datta et al., 2012; Leone et al., 2012; Kronman et al., 2014). Developing antibiotics with new antimicrobial mechanisms has again become a matter of emergency (Bassetti and Righi, 2015).

Antimicrobial peptides (AMPs) exhibit a broad antimicrobial activity independent of current drug resistance (da Costa et al., 2015). AMPs are considered as a promising solution for the drug resistance and are expected to reach a good synergistic effect when in combination with the traditional antibiotics due to their unique antimicrobial mechanism. In our previous study, we reported a novel antimicrobial peptide of S-thanatin that exhibited selective antimicrobial activity (Wu et al., 2010a,b; Bassetti and Righi, 2015). Notably, Ts showed lipopolysaccharide (LPS) binding affinity, indicating its great potential as a treatment for blood infections (Wu et al., 2010a,b). Ts was proved active against numerous Gram-negative bacteria including MDR but barely active over the Gram-positive species (Wu et al., 2011). The antimicrobial activity of Ts is structure dependent. Ts is a random coil with limited or none antimicrobial activity but adopts a beta-sheet form after activation (Wu et al., 2010a,b). Ts kills bacteria in a membrane-dependent manner. Negatively charged components such as LPS on the cell wall of Gram-negative bacteria and negatively charged lipids on bacterial cytoplastic membranes, can attract Ts electrostatically and thus promote the intercalation of Ts into the cytoplastic membrane. The cytoplastic membrane becomes leaky, which subsequently disintegrates the bacterial respiration and energization (Wu et al., 2010b).

Liposomes have been widely used as pharmaceutical carriers in the past decade due to their merits, such as reducing potential toxicity, prolonging circulation half-life *in vivo*, and enabling controlled release and active or passive targeting of specific cells, tissues, or organs (Kibria et al., 2013). Liposomes have been reported to serve as carriers for antibiotics (Furneri et al., 2000; Muppidi et al., 2011), improving the pharmacokinetics of the encapsulated antibiotics. However, to the best of our knowledge, the coupling of AMPs, such as Ts, to an antibiotic-loaded liposome and the application on bacterial cells have not been reported.

The Ts functioned liposomes were prepared by linking the Ts to the constituent lipids with a PEG linker. Ts played dual-role in this design as a targeting carrier and a bactericidin as well. The preparations were tested *in vitro* and *in vivo* for antimicrobial activities against clinical MDR isolates.

## Materials and Methods

### Chemical Reagents

Hydrogenated soybean phosphatidylcholine (HSPC), cholesterol (CHO), and 3-(N-succinimidyloxyglutaryl) aminopropyl-polyethyleneglycol (2000)-carbamyldistearoyl phosphatidyl ethanolamine (NHS-PEG2000-DSPE) were purchased from Avanti Polar Lipids (Alabaster, AL, USA). The antimicrobial peptide Ts (GSKKPVPIIYCNRRSGKCQRM) was synthesized, refolded and purified, as previously reported (Wu et al., 2010a,b). Ts was conjugated with NHS-PEG2000-DSPE using a method similar to the synthesis of RGD (arginine-glycine-aspartic acid) peptide conjugation (Kibria et al., 2013). Briefly, the Ts peptide was coupled with NHS-PEG-DSPE (1.2:1molar ratio) in deionized water at room temperature for 24 h. The conjugation was purified by HPLC using an appropriate 0–60% acetonitrile gradient in 0.1% trifluoroacetic acid, supplemented with 10 mg/ml of dihydroxy benzoic acid. The purity of the resulting product was 95% or higher. Carbonyl cyanide m-chlorophenyl hydrazone (CCCP, C9H5ClN4, CAS: 555-60-2), bis-(1,3-dibutylbarbituric acid) trimethineoxonol [DiBAC4(3)], propidium iodide (PI) and 1-(3-dimethylaminopropyl)-3-ethyl-carbodiimide (EDC) were obtained from Sigma (St. Louis, MO, USA). Carboxyl near-infrared quantum dots (QDs605) were purchased from Jiayuan QD Tech Ins (Wuhan, China).

### Microorganisms

In this study, a total of 17 clinical isolates of *K. pneumoniae* were used for the antimicrobial assay. These isolates were collected between February and May 2013 in the Center of Medical Laboratory of Zhongda Hospital (Southeast University, China). The VITEK2 system with AST-GN13 cards and GN/CE strips (bio Mérieux, Marcy l’Etoile, France) was used to confirm the identities and susceptibilities of bacteria. *K. pneumonia* ATCC 700603 from the Health Administrate of the People’s Republic of China was used as a reference.

### Animals

Adult male ICR mice (30–33 g) were obtained from the Experimental Animal Center of Yangzhou, China and reared in the Animal Environmental Control Unit under 23 ± 3°C, 50 ± 10% relative humidity, and a 12-h light-dark cycle. The animal experiments were carried out according to the guideline from the Medical Ethics Committee of Southeast University, China (Permit Number: 2013ZDSYLL109.0).

### Preparation of the Liposomes and Encapsulation Efficiency Measurement

The lipids formula, levofloxacin dosage, ammonium sulfate concentration, drug-loading temperature and time for liposome preparation were optimized. HSPC, CHO, and Ts-PEG2000-DSPE were mixed at different ratio for the Ts-LPs liposome preparation with the lipid film hydration method. Levofloxacin was loaded into liposome with the ammonium sulfate gradient method (see Supplementary Methods).

The liposome loaded levofloxacin was measured by HPLC to assess the optimization methods (Furneri et al., 2000) (see Supplementary Methods). A standard curve was made by measuring levofloxacin standard concentration series (Supplementary Figure S1). A solution containing 0-60% gradient acetonitrile supplemented with 0.1% trifluoroacetic acid was used as the mobile phase for HPLC. The injection volume was fixed to 20 μL. The results were assessed by the encapsulation efficiency (EE), which was calculated as follows: EE% = *C1/C0*^∗^100%, where *C*0 is the amount of total drug, and *C*1 is the amount of drug entrapped in the liposomes. Levofloxacin-loaded liposome without TS (LEV-LPs) was prepared in a similar way.

### Liposome Properties

The liposome size, zeta potential, and polydispersity index (PDI) of the liposome emulsion were measured using a Zetasizer Nano ZS system (Malvern Instruments Ltd, Worcestershire, UK).

The shape and size were also examined by transmission electron microscopy (TEM). The liposome suspension was placed on copper grids with films, stained with 2% (w/v) phosphotungstic acid, air-dried for 10 min, and finally examined using a JEM-1010 transmission electron microscope (JEOL, Tokyo, Japan) to determine the morphology of the liposome.

### Antimicrobial Activity Assay

MICs were used to evaluate the antimicrobial activity of Ts, levofloxacin, LPs-LEV, Ts-LPs, and Ts-LPs-LEV, according to the broth microdilution guidelines from the Clinical and Laboratory Standards Institute (CLSI) (Anonymous, 2001). LPs-LEV and Ts-LPs-LEV liposome concentrations were calculated as the levofloxacin-containing concentrations which were measured by a similar method as the EE assay. Ts-LPs concentration was calculated as the Ts-containing concentration. The final concentration for Ts, free levofloxacin, LPs-LEV or Ts-LPs-LEV in the tested wells ranged from 2 to 256 μg/mL. After incubation at 37°C for 16–24 h, the bacterial culture optical density (OD) value was measured using a microplate reader with a wavelength of 630 nm (MRX, Dynex). MICs are defined as the lowest concentration where 100% bacterial growth inhibition was reached. Each of the experiments was performed in triplicate. The combination of Ts with conventional antibiotics such as silver nitrate, ampicillin and kanamycin was tested for a synergistic effect (see Supplementary Methods).

### Bacterial Targeting of Ts and Ts-LPs

The carboxyl near-infrared quantum dot QDs605 was used to determine the bacterial targeting of Ts. QDs605 and Ts (molar ratio: 1:10) were co-incubated in a solution containing 1 mg/ml EDC for linkage for 2 h at room temperature. The prepared conjugate, Ts-QDs605 was rinsed twice with PBS (0.1M, pH7.4) and stored at 4°C before usage. The Ts-functionalized liposome containing coumarin (Ts-LPs-CM) was prepared using a similar method as described above. The cells pretreated with Ts-QDs605 or Ts-LPs-CM were imaged using a confocal microscope (Olympus, Japan) with excitation/emission wavelengths of 470/505 nm for coumarin and 388/605 nm for QDs605. The photographic parameters were chosen from default and no changes for relative measurements.

### Effects of Ts and Ts-LPs on Membrane Permeability and Potential

A single colony of *K. pneumonia* ATCC 700603 was inoculated in Luria-Bertani (LB) broth and cultured at 37°C to reach log-phase. A sample containing approximate 10^7^ cells/ml medium was prepared and added with 100 μg/ml Ts or Ts-LPs plus 10 mM CCCP (carbonyl cyanide m-chlorophenyl hydrazone, C9H5ClN4, a type of respiratory inhibitor) followed by an incubation at 37°C for 60 min. PBS was used as a negative control. The bacterial cells were retrieved and washed twice with PBS (0.1M, pH7.4). The lipophilic anionic membrane potential-sensitive dye DiBAC4(3) or nucleic acid dye PI was added at a final concentration of 10 μg/ml and incubated for 10 min at room temperature before flow cytometric analysis by BD FACS Canto (Becton Drive, NJ, USA). The excitation/emission wavelengths were 470/510 nm and 488/630 nm for DiBAC4(3) and PI, respectively. The photographic exposure time was set to 500 ms for all measurements.

### Electron Microscopy Studies of *K. pneumoniae* Treated with Ts-LPs-LEV

Transmission electron microscopy (TEM) was employed to confirm the cellular morphological changes after Ts-LPs-LEV treatment (Chapple et al., 1998). Cells were retrieved after receiving saline or Ts-LPs-LEV, and slices were made from cell pellets (see Supplementary Methods). The slices were pre-stained with aqueous uranyl acetate and lead citrate before being sent for examination with a JEM-1010 transmission electron microscope (JEOL, Tokyo, Japan).

### Drug Uptake

The accumulation of free levofloxacin and levofloxacin liposomes (LPs-LEV) was determined as previously reported (Furneri et al., 2000). The cellular levofloxacin was retrieved by destruction of the bacterial cells and measured by HPLC, as described for the liposome EE assay. The extracellular levofloxacin concentration in the supernatant was measure by HPLC as well.

### Septic Shock Model

Sixty male ICR mice were randomly grouped (15 animals in each group), and intraperitoneally inoculated with 2.5 × 10^7^ cells of clinical MDR isolate CI 130702215 of *K. pneumonia* (MIC > 256 μg/mL for levofloxacin, refer to **Table 1**). Immediately after the bacterial challenge, the animals were intravenously administrated with levofloxacin, LPs-LEV or Ts-LPs-LEV. Levofloxacin in all forms were equal at the dosage of levofloxacin of 10 mg/kg. The survival rate was monitored and blood samples were taken by tail-vein puncture every 6 h for the next 3 days.

**Table 1:** Zeta potential versus Ts-PEG2000-DSPE dosage (w/w).

*C*_lip_	*C*_T_	Zeta potential
100%	0%	-29.43 ± 1.32 mV
99%	1%	+8.56 ± 2.43 mV
95%	5%	+20.68 ± 1.72 mV

*The liposome formula was optimized as shown in Supplementary Tables S1 and S2. Ts-PEG2000-DSPE was incorporated into the formula at different concentrations to produce positively charged liposomes. The Zeta potential increased along with the concentration of Ts-PEG2000-DSPE in the liposome. At 5% (w/w) concentration, the zeta potential reached +20.68 ± 1.72 mV. *C*_*lip*_ represented the total weight ratio of HSPC:cholesterol (CHO) (7:3) used in the liposome preparation, and *C*_*T*_ was the ratio of Ts-PEG2000-DSPE.*

Approximately 24 h after bacterial challenge, 0.5 mL abdominal lavage fluid was collected using a long needle through intraperitoneal puncture at 10 min after an intraperitoneal injection of 2 mL sterile saline. Bacterial counting was performed by spreading the samples onto blood agar plates after series dilution of the samples in sterile saline. The plates were incubated overnight at 37°C for bacterial colony counting.

### Statistical Analysis

MICs are presented as the mean value from three independent measurements. The quantitative evaluations of levofloxacin accumulation in bacteria and bacterial counting in the intra-abdominal fluid and blood cultures are expressed as the means ± standard deviations (SDs). The analysis of variance (ANOVA) was used for comparison between groups. The mortality rate differences between groups were compared using Fisher’s exact test. In all the analyses, *P* < 0.05 was considered statistical significant.

## Results

### Preparation and Characterization of the Liposomes

**FIGURE 1:**
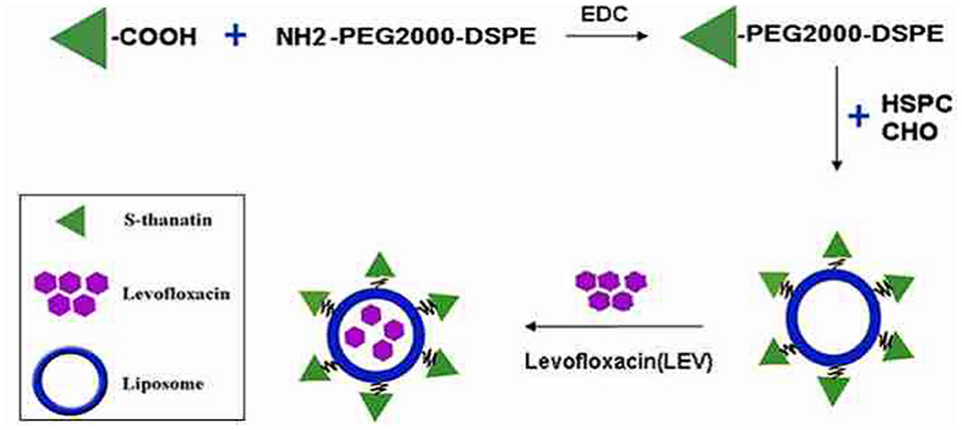
**Schematic representation of the preparation of Ts-LPs-LEV**.

**FIGURE 2:**
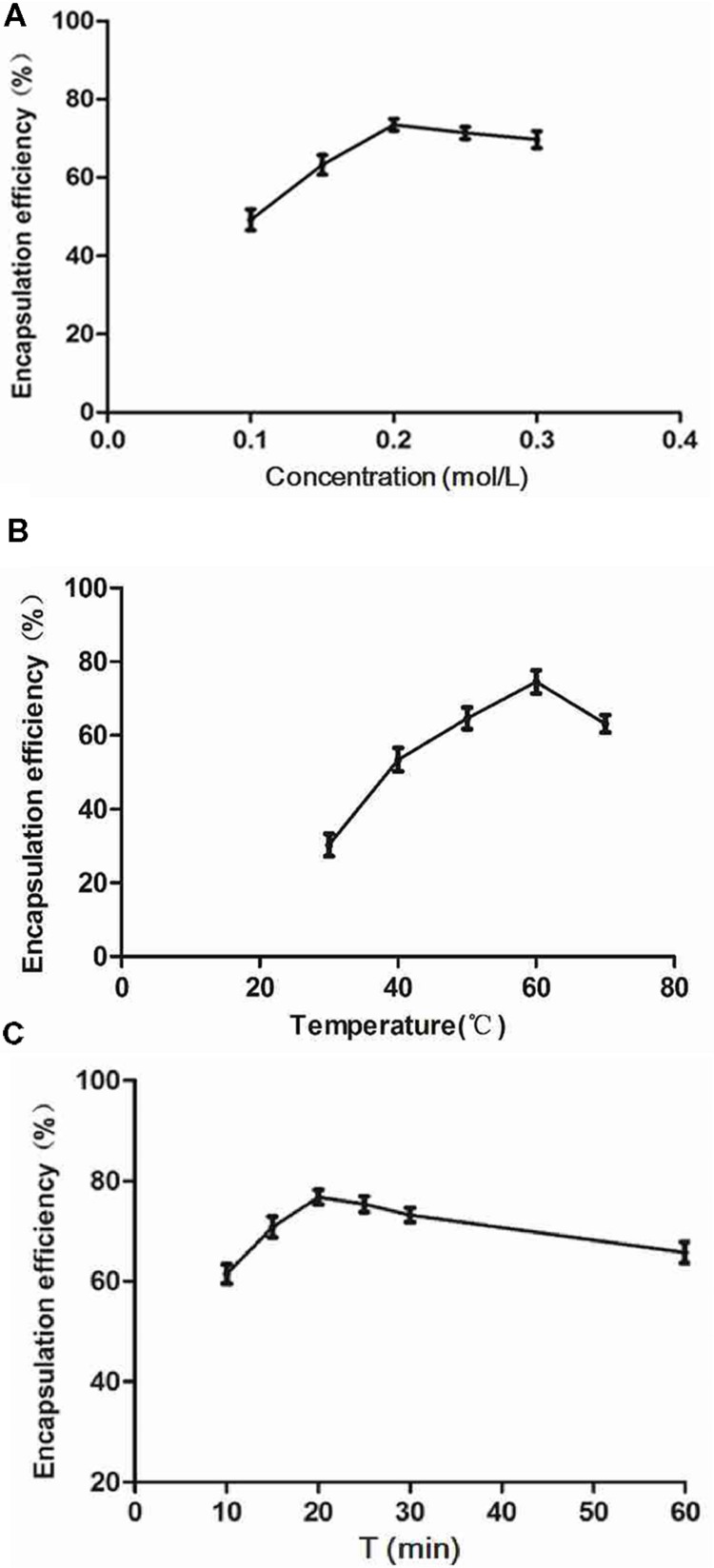
**Factors affecting the encapsulation efficiency (EE).** The drug-loading experiments were performed using the ammonium sulfate gradient method. **(A)** Influence of ammonium sulfate on EE (settings: 60°C, 30 min). **(B)** Influence of incubation temperature (settings: 0.2 mol/L ammonium sulfate, 30 min incubation). **(C)** Influence of incubation time (settings: 0.2 mol/L ammonium sulfate, 60°C). Results indicated peak EE values at 0.2 mol/L ammonium sulfate, 60°C, and an incubation time of 20 min.

**FIGURE 3:**
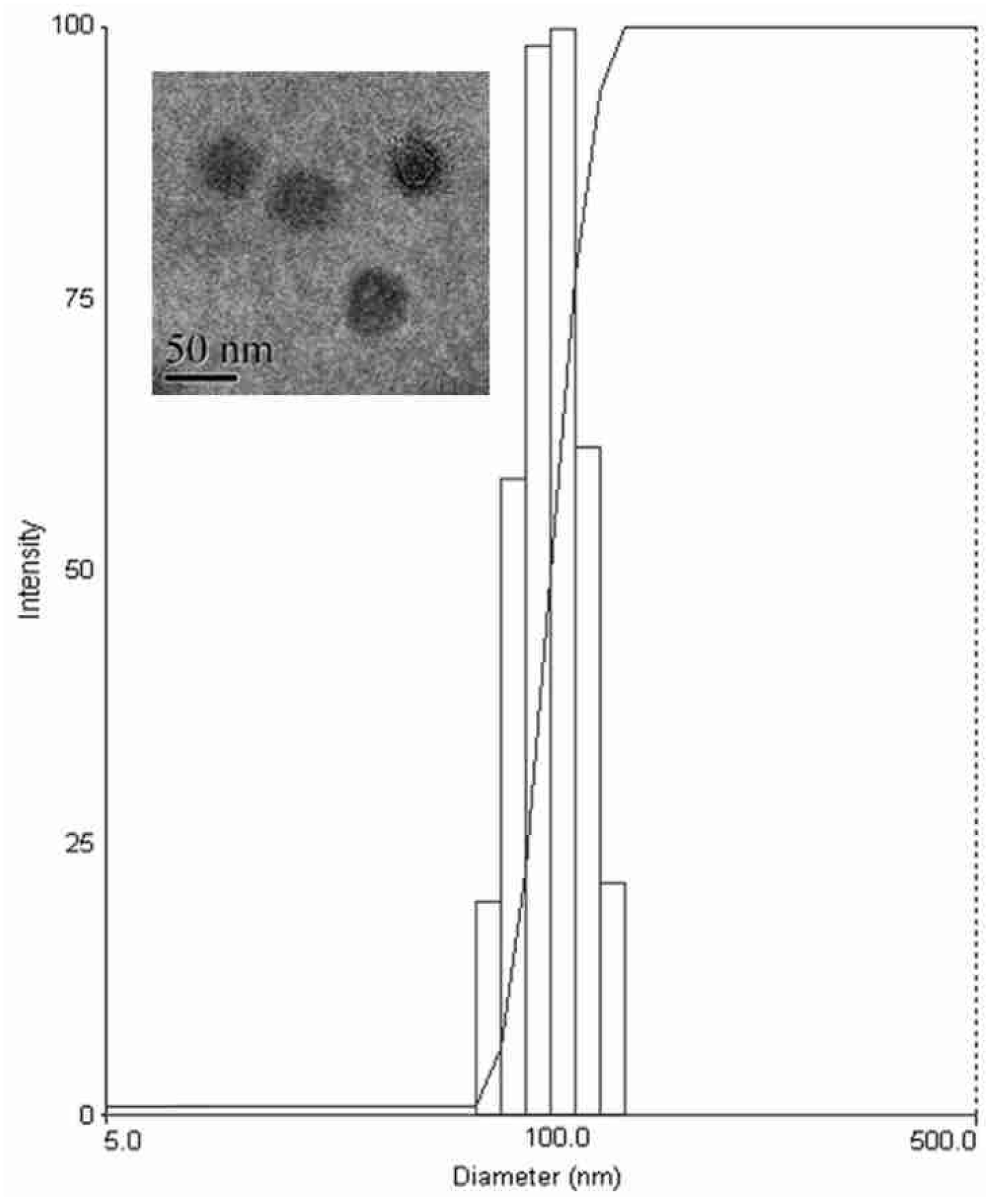
**Size distribution and transmission electron microscopy (TEM) image of Ts-LPs-LEV**.

The prepared liposome consisted of HSPC, CHO, and Ts-PEG2000-DSPE, with Ts anchored on the surface. The scheme for the preparation of Ts-LP-LEV is showed in **Figure 1**. As shown in **Table 1**, Supplementary Tables S1 and S2 and **Figure 2**, the liposome preparation of Ts-LPs-LEV was optimized as follows: (1) Formula: 30 mg/ml HSPC:CHO (4:1, w/w) plus 5% Ts-PEG2000-DSPE; (2) levofloxacin:lipids = 1:4 (mol/mol); (3) drug loading parameters: using 0.2 mol/L ammonium sulfate, incubation at 60°C for 20 min. The drug loading efficiency EE% reached 76.8 ± 2.7% after optimization. As shown in **Table 2** and **Figure 3**, the prepared Ts-LEV-LPs with a PDI of 0.254 were 152.5 ± 3.2 nm in diameter showing a good homogeneity, and were positively charged as expected. The blank liposome without levofloxacin was +20.68 mV in zeta potential. The Ts-LPs-LEV possessed a zeta potential of +4.6–5.9 mV. The positive charge indicated the presence of the positively charged Ts on the surface. The liposomes were confirmed of a spherical shape with a diameter of ∼86.9 nm by TEM (**Figure 3**). The TEM showed decreased size because the TEM imaging was performed in a high-vacuum environment causing liposome shrinkage. The prepared liposomes showed a very good stability in aqueous buffer. No apparent changes or degradation were observed after the liposomes were stored for 2 months (data not shown).

**Table 2:** Liposome properties by ZetasizerNano ZS system.

Liposome	Diameter size (nm)	PDI	Zeta potential (mV)
LPs	161.3 ± 4.5	0.293	-29.43
Ts-LPs	151.1 ± 2.4	0.216	+20.68
Ts-LPs-LEV	152.5 ± 3.2	0.254	+5.3

*A volume of 10 μL liposome sample mixed with 990 μL saline was placed in the cuvette and equilibrated for 10 min before measurement at 25°C. The result was the mean value taken from three repeats for each measurement.*

### Confocal Laser Scanning Microscopy (CLSM)

**FIGURE 4:**
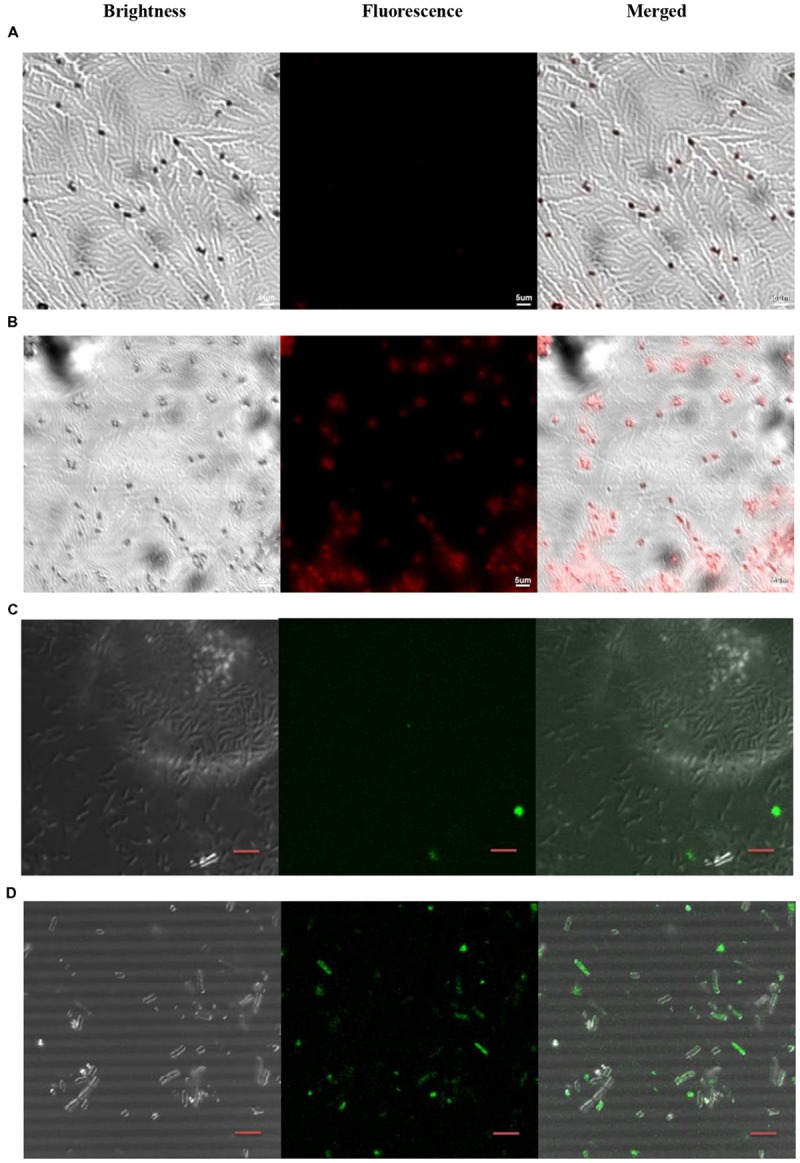
**Ts-QDs605 and Ts-LPs-CM selectively targeting *Klebsiella pneumoniae* cells.** The cell-targeting of Ts to *K. pneumoniae* ATCC 700603 observed by confocal laser scanning microscopy (CLSM) using quantum dots (QD) or coumarin (CM)-loaded liposomes (LPs): **(A)** QDs605. **(B)** Ts-QDs605. **(C)** LPs-CM. **(D)** Ts-LPs-CM. *K. pneumonia* ATCC 700603 cells (approximately 1 × 10^5^ bacteria/ml) were first incubated with Ts-QDs605 (10 nM) for 2 h at 37°C followed by trice rinse with PBS, and then placed on slides. A solution containing 4% (wt/vol) paraformaldehyde was added for sample fixation for 30 min. Ts-LPs-CM were used at 20 nM but different from Ts-QDs605 assay, the cells receiving Ts-LPs-CM were sent to CLSM without washing with PBS. The bar indicated scale of 5 μm.

Ts was labeled with the near-infrared quantum dots to confirm the bacterial uptake of Ts. Bacteria were co-incubated with Ts-QDs605 for 60 min before being sent for microscope. The CLSM results indicated that bacteria received Ts-QDs605 gave out stronger fluorescence than that of bacteria receiving QDs605 (**Figures 4A,B**). The uptake of Ts-LPs in the bacteria was also assessed by CLSM using coumarin (CM)-loaded liposome. As shown in **Figures 4C,D**, Ts-LPs-CM treated bacterial cells exhibited higher fluorescence intensity than in the LP-CM treated cells.

### Effects of Ts and Ts-LPs on Membrane Permeability and Potential by Flow Cytometric Determination

**FIGURE 5:**
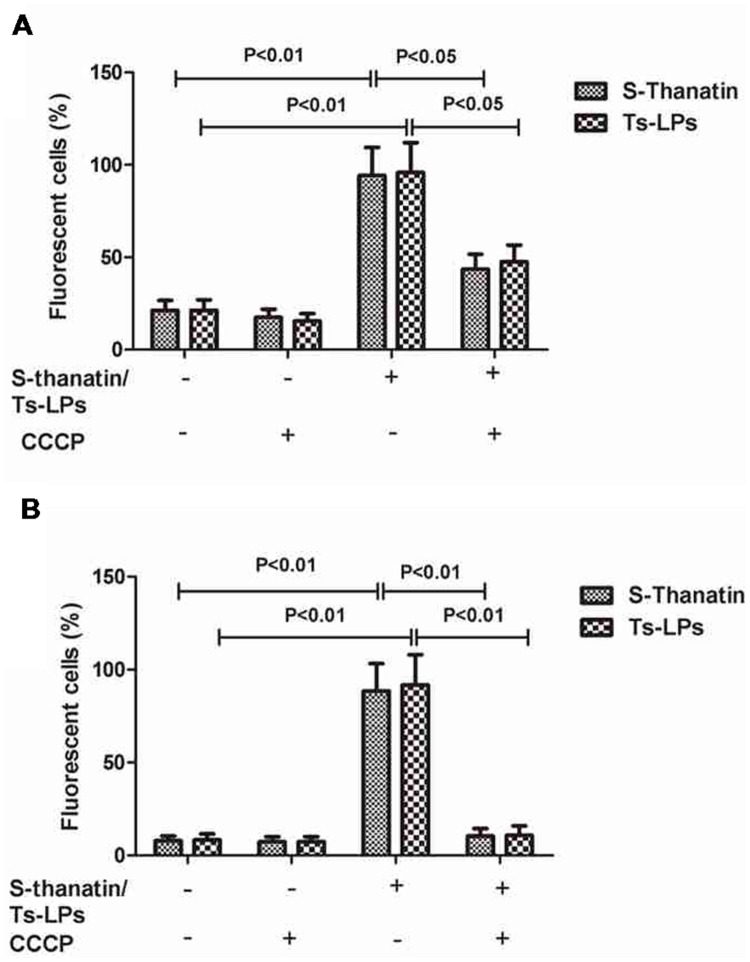
**Ts and Ts-LPs affecting membrane permeability of *K. pneumoniae* ATCC 700603.** Bacteria incubated with Ts or Ts-LPs-LEV following by staining with **(A)** PI or **(B)** DiBAC4(3). The error bars represent SD (*n* = 5).

Flow cytometry was employed to investigate the effects of Ts and Ts-LPs on the membrane permeability and potential. The results of the bacteria exposed to PI or DiBAC4(3) were shown in **Figure 5**. After adding Ts or Ts-LPs, the cell percentages of the dye-associated fluorescence significantly increased (*P* < 0.01). Bactericidal kinetics experiment indicated Ts and Ts-LPs had a rapid bactericidal effect and killed 99% bacteria within the first 10 min (Supplementary Figure S2). The CCCP with a capacity of reducing energization and respiration in cells dramatically decreased Ts- or Ts-LPs-induced uptake of PI (*P* < 0.05) and DiBAC4(3) (*P* < 0.01; **Figures 5A,B**).

### Bacteria Uptake of Levofloxacin

**FIGURE 6:**
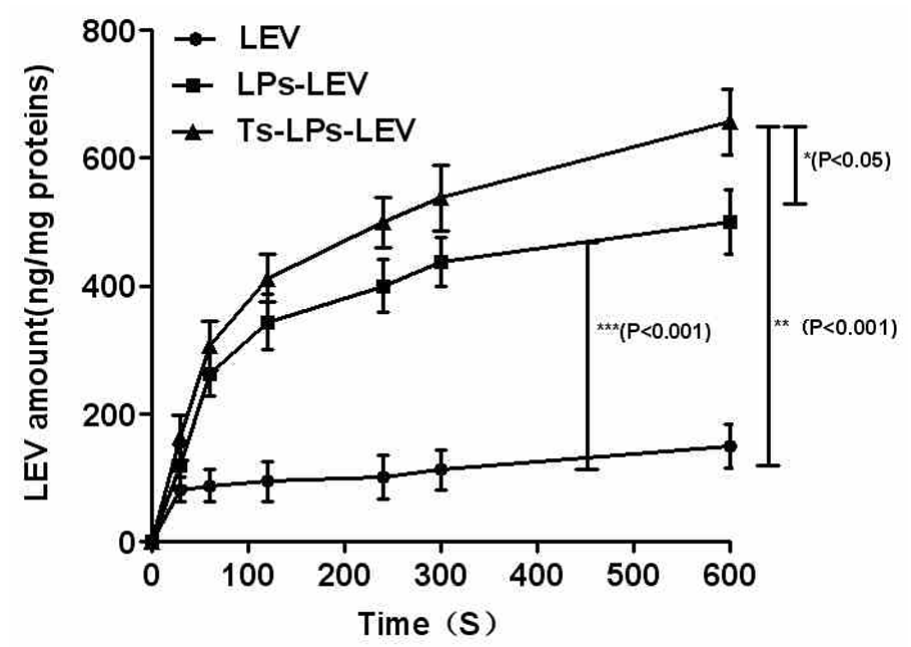
**Intra-bacterial levofloxacin accumulation of levofloxacin in *K. pneumoniae* ATCC 700603.** Error bars represent the standard deviation of three measurements.

HPLC was used to measure the intracellular levofloxacin level. The levofloxacin accumulation in different groups was presented in **Figure 6**. A gradual uptake of levofloxacin following a rapid accumulation was observed in the LPs-LEV and Ts-LPs-LEV treated groups. No apparent uptake was observed after 10 min (data not shown), and Ts and its conjugates killed 99% bacteria within 10 min by CFU counting assay (Supplementary Figure S2). A significant difference was observed between the Ts-LPs-LEV and LPs-LEV groups with intracellular concentrations of 657 ± 47 and 500 ± 52 ng/mg protein, respectively (*P* < 0.05). Compared with the free drug group (150 ± 35 ng/mg protein), levofloxacin in liposome formulations reached significantly higher intracellular accumulations (*P* < 0.001).

### *In Vitro* Antimicrobial Activities Against Clinical MDR-isolates

All 17 *K. pneumoniae* clinical isolates were multi-drug-resistant, and their MICs to levofloxacin ranged from 8 to >256 μg/mL. Their *in vitro* susceptibilities to Ts, free levofloxacin, LPs-LEV and Ts-LPs-LEV were listed in **Table 3**. The application of liposome as a drug carrier greatly improved the efficacy of levofloxacin, and incorporation of Ts again improved the efficacy of the LPs-LEV liposome. The MICs of the LPs-LEV and Ts-LPs-LEV were 1-2- and 2-16-dilution lower than that of the free drug, respectively. Drug-free Ts-LPs showed a decreased efficacy compared to Ts. No antimicrobial activity was observed in the blank liposome (data not shown). Ts showed at least additive effects when in combination with the conventional antimicrobial agents. Levofloxacin in combination with 0.2X MIC of Ts reached a synergistic effect (Supplementary Table S3).

**Table 3:** The susceptibilitiesof to *Klebsiella pneumoniae* standard strain and clinical isolates to LEV, LPs-LEV, and Ts-LPs-LEV.

Strains and clinical isolate no^a^.	Source	AMP	SAM	TZP	CFZ	CTT	CRO	CAZ	FEP	ATM	ETP	AMI	IPM	GEN	TOB	CIP	NIT	SMZ^b^	MICs (μg/ml)^c,#^				
																			LEV	LPs-LEV	Ts-LPs-LEV^d^	Ts	Ts-LPs^e^
ATCC 700603		R	I	S	S	S	S	R	S	R	S	I	S	I	I	S	S	I	8	<2	<2	8	32
CI 130218317	Drainage fluid	R	R	R	R	S	R	S	S	R	S	S	S	R	I	R	R	R	>256	128	16	32	256
CI 130322206	Sputum	R	R	S	R	R	R	R	I	R	S	S	S	R	I	S	R	R	16	8	4	16	–
CI 130130102	Secretion	R	R	R	S	S	R	R	R	R	S	S	S	R	R	R	R	S	32	16	8	16	–
CI 130205205	Blood	R	S	R	S	R	S	R	S	S	R	S	S	S	R	R	S	R	16	8	4	16	–
CI 130305114	Secretion	R	R	S	R	S	R	R	R	R	S	R	S	R	I	R	S	R	64	16	8	32	–
CI 130400215	Sputum	R	S	S	R	R	R	S	R	R	R	R	S	R	R	R	R	R	8	4	2	8	–
CI 130401230	Sputum	R	R	R	S	R	R	S	R	S	R	R	R	S	S	R	S	R	128	32	8	16	–
CI 130411303	Throat swab	R	S	S	S	R	S	R	S	S	S	S	S	R	R	S	R	S	128	64	8	16	–
CI 130411422	Sputum	R	R	S	R	S	S	R	R	S	R	S	S	S	S	R	S	S	16	8	2	8	–
CI 130501235	Secretion	R	R	S	I	R	S	S	R	S	S	S	S	S	S	S	S	S	16	16	4	16	–
CI 130612312	Sputum	R	R	R	R	S	R	R	R	R	S	S	S	S	S	R	S	R	64	16	4	16	–
CI 130615203	Sputum	R	S	R	S	R	S	S	S	S	S	S	S	R	S	S	I	S	16	8	4	8	–
CI 130702215	Sputum	R	S	S	R	S	S	R	R	R	S	R	S	R	I	S	I	R	>256	64	16	32	–
CI 130814222	Sputum	R	S	S	R	R	S	S	R	R	R	S	R	R	S	S	R	R	64	32	4	16	–
CI 130830204	Sputum	R	R	S	R	R	S	R	R	R	S	R	R	R	R	R	S	S	64	16	4	16	–
CI 130931234	Secretion	R	S	S	R	S	R	R	R	S	S	R	S	R	R	S	R	S	128	64	8	16	–
CI 130901155	Secretion	R	R	R	I	R	R	R	R	R	R	R	R	R	R	R	I	R	16	8	4	8	–

*^a^ATCC, American type culture collection; CI, clinical isolate. ^b^R, resistant; S, sensitive; I, intermediate; —, not determined; AMP, ampicillin; SAM, ampicillin–sulbactam; TZP, piperacillin–tazobactam; CFZ, cefazolin; CTT, cefotetan; CRO, ceftriaxome; CAZ, ceftazidime; FEP, cefepime; ATM, aztreonam; ETP, ertapenem; AMI, amikacin; IPM, imipenem; GEN, gentamicin; TOB, tobramycin; CIP, ciprofloxacin; NIT, furadantin; SMZ, cotrimoxazole; LEV, levofloxacin. ^c^Free drug or LEV concentration entrapped in liposomes. ^d^Ts:LEV = 1:1(w/w). ^e^Ts-LPs was calculated as the Ts-containing concentration. LPs-LEV and Ts-LPs-LEV liposome concentrations were calculated as the levofloxacin-containing concentrations. ^#^We used two times dilution method to determine the MIC values, and the MICs were from triple replicates. In most of the cases, the MICs from three replicates were the same, so there were no SD values.*

### The Bactericidal Effect by Transmission Electron Microscope

**FIGURE 7:**
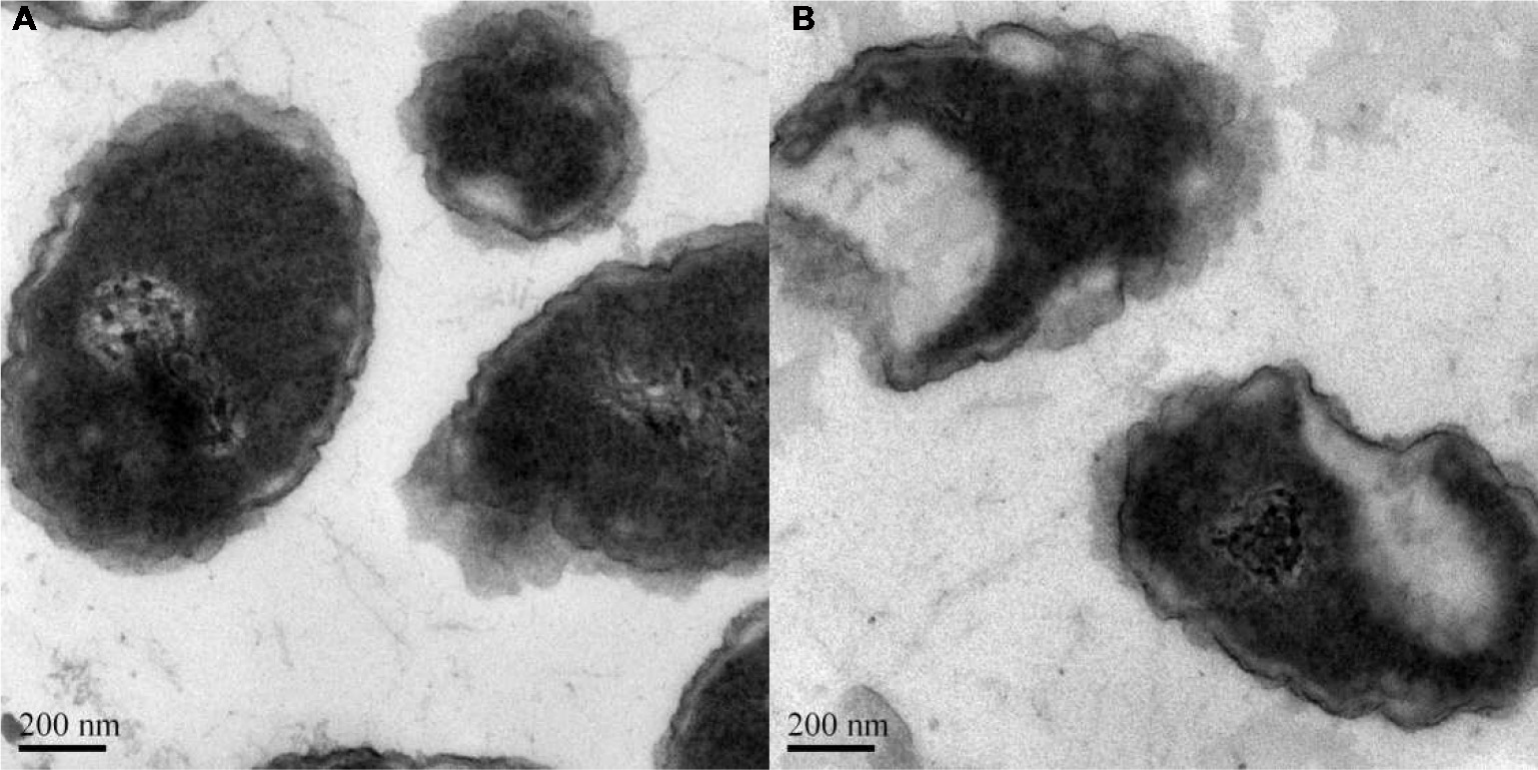
**Bactericidal effect of Ts-LPs-LEV.** Electron micrographs of *K. pneumoniae* ATCC 700603 incubated for 1 h after receiving **(A)** saline or **(B)** Ts-LPs-LEV. The length of the scale bar in the TEM image is 200 nm.

Log-phase bacterial cells were exposed to the liposome for 1 h to characterize the antimicrobial effect of Ts-LPs-LEV. Remarkable changes were observed with electron microscope (**Figure 7**) such as chaotic membrane morphology, vacuolization, chromatin concentration, and cell debris that was similar to the effects of Ts as we reported before.

### *In Vivo* Antimicrobial Activities

**FIGURE 8:**
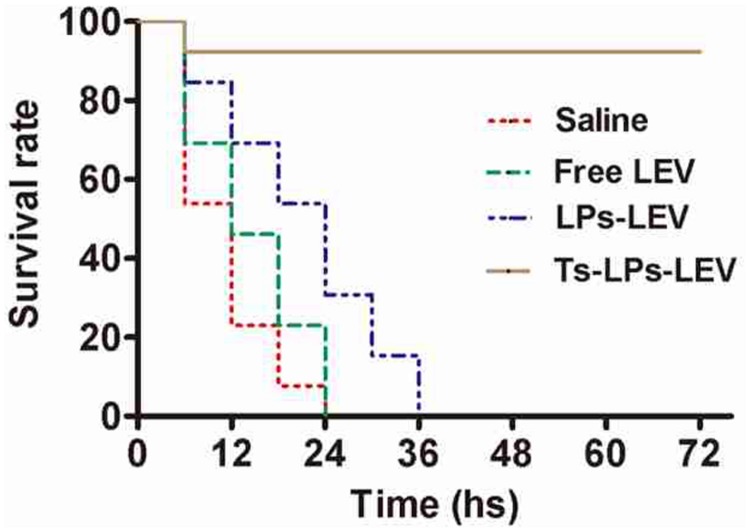
**Mortality data reported as Kaplan–Meier curves for levofloxacin formulations in a septic shock model induced by a multidrug resistant (MDR) clinical isolate.** After bacterial challenge by injection of 2.5x10^7^ CFU of the multidrug-resistant clinical isolate *K. pneumoniae* CI 130702215, the animals were immediately injected in their tail veins with sterile saline, free levofloxacin, LPs-LEV or Ts-LPs-LEV. The survival rate was monitored every 6 h for 3 days without any intervention except for the experimental drugs.

A septic shock model was established by intraperitoneal injection of MDR clinical isolate and treatment with the free drug, LPs-LEV or Ts-LPs-LEV, respectively. The lethality rate at 24 h was 100% in both the saline-treated group and free levofloxacin-treated group, whereas it was 73.3 or 6.7% for the LPs-LEV-treated group or for the Ts-LPs-LEV-treated group, respectively. All the animals from the LPs-LEV-treated group were dead within 36 h, whereas 93.3% of the animals from the Ts-LPs-LEV-treated group survived at 72 h (**Figure 8**).

The bacterial culture results were showed in **Table 4**. At 24 h, all animals were positive for bacterial culture of the blood and peritoneal fluid samples, but colony count in the groups treated with LPs-LEV and Ts-LPs-LEV was significantly less than that of the saline and levofloxacin treated groups (*P* < 0.05). The Ts-LPs-LEV showed an improved efficacy on bacteria clearance compared to LPs-LEV (*P* < 0.05). To our surprise, levofloxacin used alone showed none priority over saline to bacterial clearance (*P* > 0.2).

**Table 4:** Bacterial clearance and mouse survival in septic shock models.

Treatment	Lethality [dead/total (%)]	Bacterial count in blood (CFU/mL)	Bacterial count in peritoneal fluid (CFU/mL)
Saline	15/15 (100)	5.1 ± 2.0 × 10^6^	7.8 ± 3.1 × 10^7^
Free LEV	15/15 (100)	4.7 ± 1.5 × 10^6^	6.3 ± 2.5 × 10^7^
LPs-LEV	11/15 (73.3)	2.7 ± 1.1 × 10^3∗^	2.2 ± 1.0 × 10^4∗^
Ts-LPs-LEV	1/15 (6.7)^∗#^	1.4 ± 0.4 × 10^1∗#^	5.6 ± 1.3 × 10^1∗#^

*The samples were collected at 24 h or immediately after the dead mouse was observed. Mice were observed every 6 h. Error is represented as the standard deviation (*n* = 15). ^∗^*P* < 0.05 vs. the saline or the free LEV groups. ^#^*P* < 0.05 vs. the LPs-LEV group.*

## Discussion

The designed liposome Ts-LPs was stable and met multiple requirements for a drug carrier. The liposome properties were closely affected by lipid species and their composition. CHO is the most comprehensive example. CHO comprises up to 50% of the total lipids of mammal cell membrane but rarely present in bacterial cells. CHO renders the lipid bilayer flexibility as well as stability. Incorporation of CHO into liposome formula can reduce unfavorable liposomal leakage and prohibit membrane fusion in artificial systems (Shimanouchi et al., 2009). Charge on the liposome surface prevents self-contact and self-fusion improving the stability of the liposome. A proper charge on the liposome surface introduced by Ts was another guarantee that the produced liposome remained stable for at least 2 months, and allowed the electrostatic attraction between the liposomes with negatively charged components from the bacterial membrane. The incorporation of DSPE and HSPC was meant to stabilize the liposome and to mediate the fusion of the liposome with bacterial membranes (Ma et al., 2010). PEGylation is a mature technique and has been widely applied in pharmaceutical industry. PEGylation reduced non-specific interactions between drugs with proteins and cells, and thus decreases the drug degradation and prolongs the drug circulation in the bloodstream (Anonymous, 2007). The drug metabolism and pharmacokinetics of the liposomes would be an interesting topic for the next step.

Levofloxacin was loaded to the liposomes using the ammonium sulfate gradient method (Haran et al., 1993) and reached a high EE. The carrier capacity is an important parameter to evaluate a drug delivery system for effective antibacterial chemotherapy. The ammonium sulfate gradient method has several advantages, including a shorter preparation period, milder conditions and higher drug EE than those of other methods (Fritze et al., 2006). Our results demonstrated that the levofloxacin EE (%) of Ts-LPs-LEV was ∼76% using the ammonium sulfate gradient method, which was higher than that of the reverse phase evaporation method, ethanol injection method, or citric acid gradient method (data not shown).

Ts and levofloxacin combination against *K. pneumoniae* showed at least additive effects and it reached synergism when levofloxacin was used in combination with 0.2X MIC of Ts (Supplementary Table S3). Similarly, the antimicrobial activity assay demonstrated that the MICs of Ts-LPs-LEV against 17 *K. pneumoniae* clinical isolates were 2–16-fold lower than that of the free drug (**Table 3**). This was consistent with the drug uptake results, and was attributed to the distinguishing mechanism of action between Ts and Levofloxacin that we would discussed later. It was reasonable that Ts showed decreased activity when it was immobilized onto the liposome because such immobilization reduced the flexibility and geometrically prohibited the interaction between Ts monomers which was called self-promoting interaction during the unique membrane intercalation process of AMPs (Prado Montes de Oca, 2013). Different *K. pneumoniae* strains varied in susceptibility to Ts. It may be due to the modification at the cell envelope of wild type bacteria after receiving antibiotic treatment, particularly the LPS and/or negatively charged content. The selectivity of AMPs is largely dependent on the electrostatic interaction. Such a modification on the charge content at the cell envelope altered the susceptibility of the bacteria to Ts. However, the measurement of cellular charge of bacterial cells seemed infeasible. We tried several times but got totally different results for different cultures from the same colony. A pure study in artificial systems such as the liposome suggested the correlationship between charge and AMP efficiency (Prado Montes de Oca, 2013).

The bacterial uptake of coumarin (CM)-loaded Ts-LPs-CM was imaged by CLSM (Dong and Feng, 2007; Ma et al., 2010). The result was similar to that observed in the drug uptake assay by HPLC. The drug uptake assay revealed that the liposome delivery system was very effective. The liposome benefited the drug uptake by bacteria, and Ts could improve such effect resulting into relative higher uptake by bacteria for Ts-LPs-LEV/Ts-LPs-CM. Theoretically, Ts-LPs would result into a more promising effect if it was used *in vivo* where Ts-LPs could distinguish mammal cells from bacteria while the liposome without Ts might be distracted by mammal cells. In view of our previous study, highly positively charged Ts could selectively target bacterial cells through electrostatic interaction with negatively charged components present on the outer bacterial membranes, i.e., saccharide moieties of various natures, phospholipids, glycosphingolipids, peptidoglycan, and in particular the LPS that was proved to show affinity with Ts by ELISA assay (Wu et al., 2010b). By contrast, Ts showed a very limited cytotoxicity on mammal cells (Wu et al., 2010b).

The HPLC might not be a proper method to determine the cellular drug uptake. The Ts intercalated into the cellular membrane linking/fusing the liposomes with the cells. So it is impossible to separate the Ts-LPs from the cells by centrifugation or dialysis. Therefore, the HPLC measured the drug both inside the cells and in the surface adherent liposomes. We could not determine whether the drug remained in the liposomes or entered into the cells. However, fusion or linkage between the liposomes and the cellular membrane would greatly facilitate the drug uptake by the bacterial cells, and the drug would later enter into the cells by membrane fusion. The size of the liposome is about 100 nm while the cell is about 500–2000 nm. The CLSM image did not provide information about cellular location of the liposomes due to the limited resolution. It was hard to tell whether the liposomes entered inside or still remained at the surface. The subcellular structures of the cells are visible under electronic microscope but the electronic microscopy lacks discernment between the liposomes and cells. An image indicating the cellular location of the liposomes would be helpful to understand more details.

The *in vivo* test using mouse model after bacterial challenge indicated the multiple functions of Ts in the liposome complex. Bacterial challenge would first evoke a systematic inflammation and finally developed a multiple organ failure that killed the mice. Antibiotics treatment is not prioritized for septic patients. The serious inflammation induced by endotoxin/exotoxin is highly concerned. From the results, the merits of using Ts were very convincible. Ts resulted into a better bacterial clearance in peritoneal fluid and blood compared with free levofloxacin and LPs-LEV (*P* < 0.05; **Table 2**), and greatly improved the survival rate probably through prohibiting the LPS-mediated inflammation.

It has been suggested that transport across the gram negative bacterial cell wall might be achieved by three different ways, namely, hydrophilic transport through porin channels (Nikaido and Vaara, 1985), hydrophobic adsorption through the lipid membrane (Kotera et al., 1991) and self-promoted uptake (Chapman and Georgopapadakou, 1988). Drug properties such as the hydrophobicity, size and molecular structure can affect the hydrophilic and hydrophobic pathway. The self-promoted uptake route is based on the displacement of divalent cations from the LPSs on the bacterial outer membrane.

It is well known that the non-specific drug eﬄux and low outer membrane permeability are the major mechanisms for bacterial resistance to fluoroquinolone antibiotics (Nikaido and Vaara, 1985; Srikumar et al., 1998; Srinivasan et al., 2014). The flow cytometry results suggested that the membrane permeability of bacteria was increased by the addition of Ts or Ts-LPs. These findings indicated that Ts killed bacteria at least partially by exhausting the transmembrane potential that is closely related to the bacterial respiration and energization.

**FIGURE 9:**
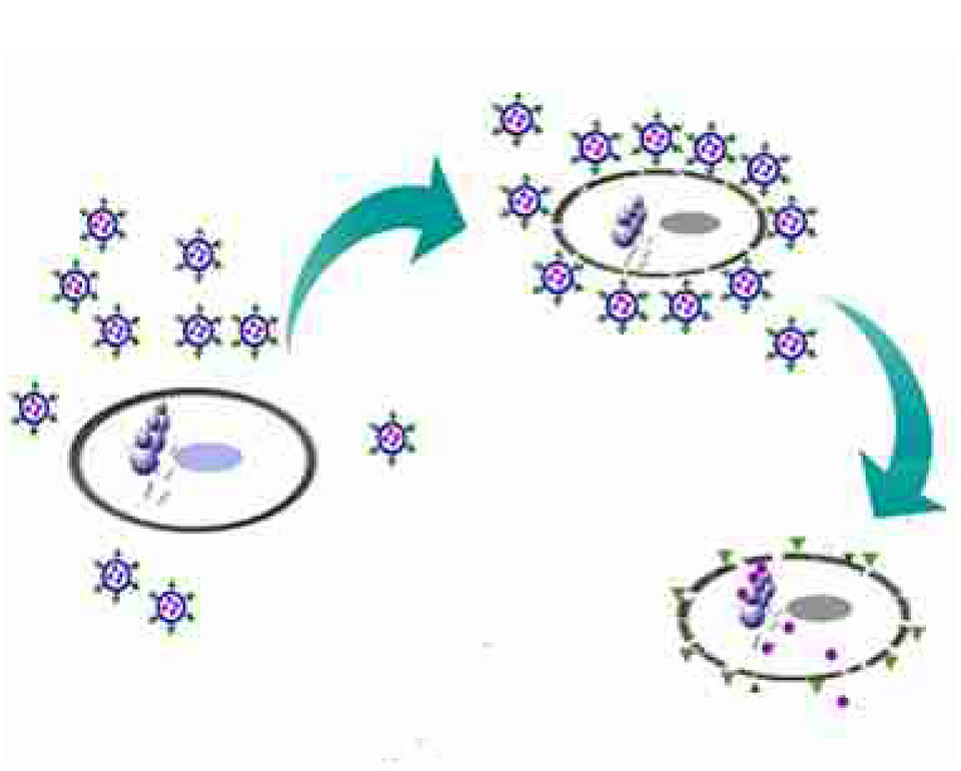
**Schematic representation of Ts-LPs-LEV killing bacteria process.** Briefly, when Ts-LPs-LEV co-incubated with the bacteria, Ts carried a large amount of levofloxacin-loaded liposomes to the bacterial surface and anchored in the outer membrane by combination with LPS and/or negatively charged components. Then, the liposomes fused with the bacterial cell outer membrane, which could enhance levofloxacin entry through hydrophobic and/or self-promoted pathway or contact release. Meanwhile, Ts perturbed the membrane lipid bilayers of the bacteria and removed the electrical potential of the membrane, affecting the activity of the drug eﬄux pumps and thus resulting in an intra-bacterial levofloxacin accumulation. The cells finally became dead and broken.

Our results are consistent with the following proposed mechanism of TS-LPs-LEV action : (1) Ts led levofloxacin-loaded liposomes to the bacterial surface by interacting with negatively charged content on the bacterial envelop such as LPS; (2) liposomes fused with the bacterial cell outer membrane, which could enhance drug entry through the hydrophobic and/or self-promoted pathway; (3) the integrity of the bacterial cytoplasmic membrane and the respiration chain located on the cytoplasmic membrane was compromised due to Ts insertion resulting into more drug uptake and active eﬄux failure; and (4) liposomes gave a contact release of levofloxacin (Furneri et al., 2000) close to the bacterial cell surface, resulting in a higher drug uptake than the free drug (**Figure 9**).

In summary, we successfully developed an antimicrobial-peptide-functionalized levofloxacin-loaded liposome (Ts-LPs-LEV). The liposome showed bacterial selectivity *in vitro* and exhibited an excellent therapeutic effect in the septic mouse model induced by MDR *K. pneumoniae* clinic isolate. We presented a very promising approach to design a novel antibiotics deliver of targeting potential. The clinical application of this promising antibiotic delivery approach deserves further exploration.

## Author Contributions

XF discussed the results, did the *in vitro* antimicrobial assays and revised the paper; JF and PW did most of the *in vivo* and *in vitro* experiments and drafted the paper; XW helped to revise the manuscript; GW was the group leader offering supervision and financial support.

## Conflict of Interest Statement

The authors declare that the research was conducted in the absence of any commercial or financial relationships that could be construed as a potential conflict of interest.

## References

[B1] Anonymous (ed). (2001). *National Committee for Clinical Laboratory Standards* (2001) Methods for Dilution Antimicrobial Susceptibility Tests for Bacteria That Grow Aerobically 5th Edn Wayne, PA: National Committee for Clinical Laboratory Standards.

[B2] Anonymous (2007). Drug discovery today: technologies (2007) volume 4 issues 3/4. *Drug Discov. Today Technol.* 4:e89–e108. 10.1016/j.ddtec.2009.07.001

[B3] Bassetti M. Righi E. (2015). Development of novel antibacterial drugs to combat multiple resistant organisms. *Langenbecks Arch. Surg.* 400 153–165. 10.1007/s00423-015-1280-4 25667169

[B4] Chapman J. S. Georgopapadakou N. H. (1988). Routes of quinolone permeation in *Escherichia coli*. *Antimicrob. Agents Chemother.* 32 438–442. 10.1128/AAC.32.4.438 3132091 PMC172197

[B5] Chapple D. S. Mason D. J. Joannou C. L. Odell E. W. Gant V. Evans R. W. (1998). Structure-function relationship of antibacterial synthetic peptides homologous to a helical surface region on human lactoferrin against *Escherichia coli* serotype O111. *Infect. Immun.* 66 2434–2440. 9596699 10.1128/iai.66.6.2434-2440.1998 PMC108221

[B6] da Costa J. P. Cova M. Ferreira R. Vitorino R. (2015). Antimicrobial peptides: an alternative for innovative medicines? *Appl. Microbiol. Biotechnol.* 99 2023–2040. 10.1007/s00253-015-6375-x 25586583

[B7] Datta S. Wattal C. Goel N. Oberoi J. K. Raveendran R. Prasad K. J. (2012). A ten year analysis of multi-drug resistant blood stream infections caused by *Escherichia coli* & *Klebsiella pneumoniae* in a tertiary care hospital. *Indian J. Med. Res.* 135 907–912. 22825611 PMC3410219

[B8] Dong Y. Feng S. S. (2007). In vitro and in vivo evaluation of methoxy polyethylene glycol-polylactide (MPEG-PLA) nanoparticles for small-molecule drug chemotherapy. *Biomaterials* 28 4154–4160. 10.1016/j.biomaterials.2007.05.026 17576004

[B9] Fritze A. Hens F. Kimpfler A. Schubert R. Peschka-Suss R. (2006). Remote loading of doxorubicin into liposomes driven by a transmembrane phosphate gradient. *Biochim. Biophys. Acta* 1758 1633–1640. 10.1016/j.bbamem.2006.05.028 16887094

[B10] Furneri P. M. Fresta M. Puglisi G. Tempera G. (2000). Ofloxacin-loaded liposomes: in vitro activity and drug accumulation in bacteria. *Antimicrob. Agents Chemother.* 44 2458–2464. 10.1128/AAC.44.9.2458-2464.2000 10952595 PMC90085

[B11] Haran G. Cohen R. Bar L. K. Barenholz Y. (1993). Transmembrane ammonium sulfate gradients in liposomes produce efficient and stable entrapment of amphipathic weak bases. *Biochim. Biophys. Acta* 1151 201–215. 10.1016/0005-2736(93)90105-9 8373796

[B12] Kibria G. Hatakeyama H. Ohga N. Hida K. Harashima H. (2013). The effect of liposomal size on the targeted delivery of doxorubicin to Integrin alphavbeta3-expressing tumor endothelial cells. *Biomaterials* 34 5617–5627. 10.1016/j.biomaterials.2013.03.094 23623323

[B13] Kotera Y. Watanabe M. Yoshida S. Inoue M. Mitsuhashi S. (1991). Factors influencing the uptake of norfloxacin by *Escherichia coli*. *J. Antimicrob. Chemother.* 27 733–739. 10.1093/jac/27.6.733 1938683

[B14] Kronman M. P. Zerr D. M. Qin X. Englund J. Cornell C. Sanders J. E. (2014). Intestinal decontamination of multidrug-resistant *Klebsiella pneumoniae* after recurrent infections in an immunocompromised host. *Diagn. Microbiol. Infect. Dis.* 80 87–89. 10.1016/j.diagmicrobio.2014.06.006 25041704 PMC4162817

[B15] Leone I. Mungo E. Bisignano G. Chirillo M. G. Savoia D. (2012). *Klebsiella pneumoniae*: emergence of multi-drug-resistant strains in Northwest Italy. *J. Infect.* 64 535–537. 10.1016/j.jinf.2012.01.009 22285203

[B16] Ma Y. Zheng Y. Liu K. Tian G. Tian Y. Xu L. (2010). Nanoparticles of Poly(Lactide-Co-Glycolide)-d-a-Tocopheryl Polyethylene Glycol 1000 Succinate Random Copolymer for Cancer Treatment. *Nanoscale Res. Lett.* 5 1161–1169. 10.1007/s11671-010-9620-3 20596457 PMC2893931

[B17] Muppidi K. Wang J. Betageri G. Pumerantz A. S. (2011). PEGylated liposome encapsulation increases the lung tissue concentration of vancomycin. *Antimicrob. Agents Chemother.* 55 4537–4542. 10.1128/AAC.00713-11 21788465 PMC3186981

[B18] Nikaido H. Vaara M. (1985). Molecular basis of bacterial outer membrane permeability. *Microbiol. Rev.* 49 1–32. 2580220 10.1128/mr.49.1.1-32.1985 PMC373015

[B19] Prado Montes de Oca E. (2013). Antimicrobial peptide elicitors: new hope for the post-antibiotic era. *Innate Immun.* 19 227–241. 10.1177/1753425912460708 23160387

[B20] Shimanouchi T. Ishii H. Yoshimoto N. Umakoshi H. Kuboi R. (2009). Calcein permeation across phosphatidylcholine bilayer membrane: effects of membrane fluidity, liposome size, and immobilization. *Colloids Surf. B Biointerfaces* 73 156–160. 10.1016/j.colsurfb.2009.05.014 19560324

[B21] Srikumar R. Kon T. Gotoh N. Poole K. (1998). Expression of *Pseudomonas aeruginosa* multidrug eﬄux pumps MexA-MexB-OprM and MexC-MexD-OprJ in a multidrug-sensitive *Escherichia coli* strain. *Antimicrob. Agents Chemother.* 42 65–71. 9449262 10.1128/aac.42.1.65 PMC105457

[B22] Srinivasan V. B. Singh B. B. Priyadarshi N. Chauhan N. K. Rajamohan G. (2014). Role of novel multidrug eﬄux pump involved in drug resistance in *Klebsiella pneumoniae*. *PLoS ONE* 9:e96288 10.1371/journal.pone.0096288 PMC4019481 24823362

[B23] Wu G. Fan X. Li L. Wang H. Ding J. Hongbin W. (2010a). Interaction of antimicrobial peptide s-thanatin with lipopolysaccharide in vitro and in an experimental mouse model of septic shock caused by a multidrug-resistant clinical isolate of *Escherichia coli*. *Int. J. Antimicrob. Agents* 35 250–254. 10.1016/j.ijantimicag.2009.11.009 20045294

[B24] Wu G. Wu H. Fan X. Zhao R. Li X. Wang S. (2010b). Selective toxicity of antimicrobial peptide S-thanatin on bacteria. *Peptides* 31 1669–1673. 10.1016/j.peptides.2010.06.009 20600431

[B25] Wu G. Q. Li X. F. Fan X. B. Wu H. B. Wang S. L. Shen Z. L. (2011). The activity of antimicrobial peptide S-thanatin is independent on multidrug-resistant spectrum of bacteria. *Peptides* 32 1139–1145. 10.1016/j.peptides.2011.03.019 21453736

