# Supplementary material for: S-thanatin functionalized liposome potentially targeting on Klebsiella pneumoniae and its application in sepsis mouse model
Source: Front Pharmacol. 2015 Oct 27;6:249. doi: 10.3389/fphar.2015.00249 (PMC4621865; doi:10.3389/fphar.2015.00249)
Supplement: Supplementary file 1 [file Data_Sheet_1.DOCX]

**Supplementary**

**Methods:**

**Liposome preparation**

A thin lipid film was dried down from 2ml lipid mixture mixed with 2 ml methanol under a nitrogen stream followed by vacuum evaporation using a rotating evaporator to completely remove the organic solvent at 30°C overnight. The lipid film was added with 4ml 0.3% ammonium sulfate solution and incubated for 30 min at room temperature with gentle shaking, followed by a 30-min (1 s/1 s interval) bath sonication (AU-25 C, Aiwa, Tokyo, Japan). The levofloxacin was loaded into liposomes by the ammonium sulfate gradient method. Briefly, the liposome suspension was dialyzed against distilled water overnight to establish an ammonium sulfate gradient. Approximately 12.5 % (w/w) levofloxacin was added to the liposome suspension and incubated at different temperature. The un-trapped levofloxacin was removed by dialysis against saline (3 kDa cellulose membrane).

**HPLC assay for Levofloxacin EE% in liposome**

Briefly, a volume of 20 μL prepared liposome was dissolved by adding 980 μL methanol-acetonitrile (8:2 [vol/vol]) and the levofloxacin concentration was measured as the total drug. A volume of 2 mL prepared liposome was dialyzed against saline buffer overnight and then adjusted to a volume of 4 mL. A volume of 40 μL dialyzed liposome was taken and mixed with 960 μL methanol-acetonitrile (8:2 [vol/vol]). The mixture was treated with water-bath ultra-sonication for 10 min and filtered before being sent to HPLC. The levofloxacin concentration after dialysis was considered as the entrapped drug.

**Measurements by Zetasizer Nano ZS system**

Briefly, a volume of 10 μL liposome sample mixed with 990 μL saline buffer was placed in the cuvette and equilibrated for 10 min before measurement at 25 degrees. The result was taken from three repeats for each measurement.

**Antimicrobial assay**

Briefly, the bacteria were grown to the logarithmic phase and diluted to 10^5^-10^6^ CFU/mL with sterilized phosphate-buffered saline (PBS). A concentration series of Ts, levofloxacin, LPs-LEV, Ts-LPs or Ts-LPs-LEVwere prepared using a two-fold dilution method. A 90-μL aliquot of the bacterial suspension was mixed with 10 μL of Ts, levofloxacin, LPs-LEV, Ts-LPs or Ts-LPs-LEV separately.

**FICI for combination test**

The fractional inhibitory concentration index (FICI) for combinations of Ts with levofloxacin was calculated according to the equation FICI = FIC_A_+ FIC_B_ = C_A_/MIC_A_+ C_B_/MIC_B_, where C_A_ and C_B_ are the MICs of A and B tested in the combination, MIC_A_ and MIC_B_ are the MICs of A and B used alone, and FIC_A_ and FIC_B_ are the fractional inhibitory concentrations (FICs) of A and B. The FICIs were interpreted as follows: <0.5, synergy; 0.5–2.0, additive; >2.0, antagonism.

**Cell preparation for TEM**

Briefly, log-phase *K. pneumoniae ATCC 700603* (approximately 10^8^ bacteria/ml) was incubated with or without Ts-LPs-LEV (500 μg/mL)for 1 h in LB.The cells were retrieved by centrifugation at 4000 g for 5 min, and then prefixed with 2.5% glutaraldehyde, followed by a postfixationwith 0.5% osmium tetroxide and a dehydration in a gradient ethanol solution. The cell pellets were retrieved and embedded in medium resin for polymerization for 24 h at 60°C. Slices of 50-nm thickness were then prepared.

**Cell preparation for drug uptake assay**

The accumulation of free levofloxacin and levofloxacin liposomes (LPs-LEV) was determined as previously reported. Briefly, *K. pneumonia* ATCC 700603 was grown in LB broth with shaking at 37°C to a logarithmic growth phase.The bacterial cells were retrieved by centrifugation at 5,600 gfor 10 min at 4°C. The cell pellets were washed twice with cold PBS (50 mM, pH 7.0) and then resuspended in45 mLPBS. The cell resuspension was added with levofloxacin, LPs-LEV, or Ts-LPs-LEV at anequal concentration of 10 mg/mL levofloxacin, and incubated at 37°C. An aliquot of 0.5 mL bacterial culture was withdrawn from each incubationat different time points (0, 30, 60, 120, 240, 300 and 600 s) and immediately transferred into a centrifuge tube containing 1 mL of coldPBS for centrifugation at 4°C for 5 min. The cell pellets were resuspended in 1 mL 0.1M glycine hydrochloride (pH 2.0) after being washed twice with cold PBS.

**Results**

Table S1 Factors and Levels of orthogonal test for liposome formula optimization

| Level | Factors | | |
| --- | --- | --- | --- |
|  | Factor A (C_HSPC_)/(mg•mL^-1^) | Factor B(LEV:LP) | Factor C(HSPC:Ch) |
| 1 | 20 | 5:1 | 2:1 |
| 2 | 30 | 10:1 | 4:1 |
| 3 | 40 | 15:1 | 8:1 |

The factors affecting the liposome preparation such as phospholipids dosage, drug to lipids ratio and HSPC to cholesterol ratio, were optimized. Three levels, namely the low, middle and high levels were set for each factor for the orthogonal test to optimize the conditions. C_HSPC_ represented the concentration of HSPC used in the liposome preparation; LEV represented levofloxacin; LP represented the total amount ofHSPC and cholesterol; Ch represented cholesterol.

Table S2Liposome formula optimization by L_9_(3^4^) orthogonal test

| Experiment No. | Factor A | Factor B | Factor C | EE (%) |
| --- | --- | --- | --- | --- |
| No.1 | 1 | 1 | 1 | 55.41 |
| No.2 | 1 | 2 | 3 | 66.28 |
| No.3 | 1 | 3 | 2 | 64.59 |
| No.4 | 2 | 1 | 2 | 72.34 |
| No.5 | 2 | 2 | 1 | 76.55 |
| No.6 | 2 | 3 | 3 | 71.42 |
| No.7 | 3 | 1 | 3 | 59.2 |
| No.8 | 3 | 2 | 2 | 67.72 |
| No.9 | 3 | 3 | 1 | 66.93 |
| K1 | 186.27 | 186.96 | 198.9 |  |
| K2 | 220.32 | 210.54 | 204.66 |  |
| K3 | 193.86 | 202.95 | 196.89 |  |
| *$\overline{K_{1}}$* | 62.09 | 62.32 | 66.30 |  |
| *$\overline{K_{2}}$* | 73.44 | 70.18 | 68.22 |  |
| *$\overline{K_{3}}$* | 64.62 | 67.65 | 65.63 |  |
| R | 11.35 | 7.86 | 2.59 |  |

The weighting coefficient for the tested factors ranked as A>B>C. The intra weighting coefficient of each level within each factors were：A) 2＞3＞1； B) 2＞3＞1； C) 2＞1＞3. The best combination was A_2_B_2_C_2_ that is 30mg/ml HSPC, 7.5 mg/ml cholesterol and 3.75 mg/ml levofloxacin.

Table S3. Ts in combination with conventional antibiotics against *K. pneumoniae* ATCC700603

| Ts dosage | MICs of agents used in the combination (µg/ml) | | | | | |
| --- | --- | --- | --- | --- | --- | --- |
|  | AgNO_3_/FIC | Amp/FIC | Van/FIC | Qui/FIC | Kan/FIC | LEV/FIC |
| 0.2X MIC | 16/1.2 | 512/1.2 | 128/1.2 | 64/0.7 | 32/0.7 | ***2/0.45*** |
| 0.4X MIC | 8/0.9 | 512/1.4 | 64/0.9 | 64/0.9 | 8/0.0.53 | 2/0.65 |
| 0.6X MIC | 2/0.73 | 128/0.85 | 64/1.1 | 32/0.85 | 8/0.73 | 1/0.73 |

The MICs of the indicated agents were tested in combination with a constant concentration of 0.2X, 0.4X or 0.6X MIC of Ts against *K. pneumoniae* ATCC700603. Amp, Van, Qui, Kan and LEV represented ampicillin, vancomycin, quinolone, kanamycin and levofloxacin, respectively. The MICs of AgNO_3,_ vancomycin, quinolone, kanamycin and levofloxacin against *K. pneumoniae* ATCC700603 were 16, 512, 128, 128, 64 and 8 µg/ml, respectively.

Figure S1 standard curve of levofloxacin. The concentration vs. absorbance exhibited a linear relationship at concentrations between 2 and 14 μg/ml which covered the range of our measurement for EE%. y = 0.0747x -0.0464, R^2^ = 0.9997

Figure S2 Bactericidal kinetics of Ts and Ts-LPs. Fresh medium pre-inoculated with 2x10^5^ CFU/ml *K. pneumonia* ATCC 700603 was added with 4xMIC of Ts or Ts-LPs and then incubated at 37 degrees. A 10 μl aliquot was retrieved from the incubation every 2 min and diluted into 1 ml fresh medium. The dilution was drawn on plate dish containing LB agar medium, and then incubated overnight before the CFU counting.
